# Using big data from health records from four countries to evaluate chronic disease outcomes: a study in 114 364 survivors of myocardial infarction

**DOI:** 10.1093/ehjqcco/qcw004

**Published:** 2016-02-15

**Authors:** Eleni Rapsomaniki, Marcus Thuresson, Erru Yang, Patrick Blin, Phillip Hunt, Sheng-Chia Chung, Dimitris Stogiannis, Mar Pujades-Rodriguez, Adam Timmis, Spiros C. Denaxas, Nicolas Danchin, Michael Stokes, Florence Thomas-Delecourt, Cathy Emmas, Pål Hasvold, Em Jennings, Saga Johansson, David J. Cohen, Tomas Jernberg, Nicholas Moore, Magnus Janzon, Harry Hemingway

**Affiliations:** 1 Farr Institute of Health Informatics Research, University College London, London, UK; 2 Statisticon AB, Uppsala, Sweden; 3 Retrospective Observational Studies, Evidera, Lexington, MA, USA; 4 Department of Pharmacology, CIC Bordeaux CIC1401 INSERM, University of Bordeaux, Bordeaux, France; 5 Department of Mathematics, National and Kapodistrian University of Athens, Athens, Greece; 6 Hôpital Européen Georges Pompidou, Paris, France; 7 Epidemiology, AstraZeneca Rueil-Malmaison, Rueil-Malmaison, France; 8 Real World Evidence, AstraZeneca Luton, Luton, UK; 9 Medical Department, AstraZeneca Nordic-Baltic, Oslo, Norway; 10 Global Payer Evidence and Pricing, AstraZeneca R&D, Cambridge, UK; 11 Global Medicines Development, AstraZeneca Gothenburg, Mölndal, Sweden; 12 Saint Luke's Mid America Heart Institute, Kansas City, MO, USA; 13 Department of Medicine, Karolinska Institutet, Huddinge, Sweden; 14 Department of Cardiology, Karolinska University Hospital, Stockholm, Sweden; 15 Department of Cardiology and Department of Medical and Health Sciences, Linköping University, Linköping, Sweden

**Keywords:** Acute myocardial infarction, Co-morbidities, Healthcare systems, International comparison, Long-term outcomes, EHR

## Abstract

**Aims:**

To assess the international validity of using hospital record data to compare long-term outcomes in heart attack survivors.

**Methods and results:**

We used samples of national, ongoing, unselected record sources to assess three outcomes: cause death; a composite of myocardial infarction (MI), stroke, and all-cause death; and hospitalized bleeding. Patients aged 65 years and older entered the study 1 year following the most recent discharge for acute MI in 2002–11 [*n* = 54 841 (Sweden), 53 909 (USA), 4653 (England), and 961 (France)]. Across each of the four countries, we found consistent associations with 12 baseline prognostic factors and each of the three outcomes. In each country, we observed high 3-year crude cumulative risks of all-cause death (from 19.6% [England] to 30.2% [USA]); the composite of MI, stroke, or death [from 26.0% (France) to 36.2% (USA)]; and hospitalized bleeding [from 3.1% (France) to 5.3% (USA)]. After adjustments for baseline risk factors, risks were similar across all countries [relative risks (RRs) compared with Sweden not statistically significant], but higher in the USA for all-cause death [RR USA vs. Sweden, 1.14 (95% confidence interval 1.04–1.26)] and hospitalized bleeding [RR USA vs. Sweden, 1.54 (1.21–1.96)].

**Conclusion:**

The validity of using hospital record data is supported by the consistency of estimates across four countries of a high adjusted risk of death, further MI, and stroke in the chronic phase after MI. The possibility that adjusted risks of mortality and bleeding are higher in the USA warrants further study.

## Introduction

Health records from different health systems might provide insights into the care of patients with chronic diseases and the long-term outcomes of these conditions,^1,2^ but there have been few comparisons across countries. National hospital data are collected and coded in health systems in many countries and such data (compared with voluntary registries or consented studies) may provide samples that are larger, more nationally representative, and not limited to the study of any one disease, or any one stage of its development.^3^ However, there are important concerns about the quality and validity of such data.

In coronary disease, most studies of outcomes following myocardial infarction (MI) have focused on the acute phase post-MI, typically up to 1 year. However, given marked improvements over the past decade in short-term and long-term mortality following MI,^4–6^ there is a growing need to characterize the outcomes experienced by patients in whom follow-up begins after the acute phase. By the time of the first anniversary following admission for an acute MI, dual antiplatelet therapy,^7–10^ cardiac rehabilitation, and cardiologist follow-up^11^ have commonly ended, and uptake of secondary prevention medication may be declining.^12^ Recent clinical guidelines^7–10^ do not directly address the care of patients in this chronic phase of disease, whereas a recent trial found that prolonged dual antiplatelet therapy beyond the first year after an acute MI lowers the risk of cardiovascular death, MI, and stroke.^13^

To deliver better long-term care for patients surviving MI, two central questions need addressing. First, what is the risk of major clinical outcomes following the high-risk acute post-MI phase? Nearly all previous studies^14,15^ of MI outcomes start in the acute hospital setting rather than in the community, and it is well known that early events predominate in estimates of long-term risk. Most of the information on long-term outcomes available so far is derived from trials and voluntary registries, whose risks may not extrapolate to the wider patient population.^16^ Secondly, how do long-term clinical outcomes vary in different health systems? While international comparisons of cancer outcomes^17^ have influenced policy and quality-improvement initiatives, in coronary disease comparisons have been limited to the acute hospital care setting.^5,18,19^

To answer these questions, we sought national, unselected, ongoing sources of data provided by the health systems in four countries. While these data sources have been used for acute MI outcomes research within countries,^20^ their use in evaluations of the chronic phase of disease has been much less common, and the present study is the first to use such data to compare outcomes between the USA and European countries (Sweden, England, and France). Our objective was to test the validity of using such hospital record data to estimate and compare across countries the risk of three prognostic outcomes among MI survivors: all-cause death; composite of MI, stroke, or all-cause death; and hospitalized bleeding.

## Methods

### Health record data sources and study population

We analysed anonymized patient data from national ongoing hospital sources that use the International Classification of Diseases (ICD) coding system. In Sweden, we used nationwide (100% population coverage) administrative linked data (not directly used for reimbursement) obtained from mandatory Swedish national registries: the National Inpatient Register, the Swedish Prescribed Drug Register, and the Cause of Death Register. In the USA, we used an administrative claims database (Medicare) obtained from the Centers for Medicare & Medicaid Service's standard analytic files that are publicly available; these contain a nationally representative 5% random sample of all Medicare beneficiaries, based on selecting records with 05, 20, 45, 70, or 95 in positions 8 and 9 of the Social Security Number (SSN) (Centers for Medicaid and Medicare, Standard Analytical Files. https://www.cms.gov/research-statistics-data-and-systems/files-for-order/limiteddatasets/standardanalyticalfiles.html, accessed 17 December 2015). Patients are linked across the enrolment and eligibility file and service claims files using a unique encrypted SSN. Deaths are determined by linkage to the National Death file. In England, a single primary care electronic health record (EHR) covers >95% of the population and we used a 4% sample available for research. We used the CALIBER research platform of primary care EHRs (Clinical Practice Research Datalink), linked via the unique identifier of the National Health Service number with other record sources [the Myocardial Ischaemia National Audit Project (MINAP), the Hospital Episodes Statistics database, and the nationwide cause-specific mortality database]. The CALIBER data resource has been shown to be representative of the general population,^21–23^ and valid for cardiovascular research.^24–28^ In France, the source data came from the administrative claims insurance database, which covers 95% of the French population. The sample [Echantillon Généraliste des Bénéficiaires (EGB)] available for researchers was built by randomly selecting patients from their national id check number (97 random possibilities). This permanent 1/97 sample has been shown to be representative in terms of age, sex, social status, and overall medical expenses.^29–33^ The EGB health insurance claims data are linked to hospital discharge summaries and death registry through the unique healthcare identifier number.

Our study population was defined by the presence of three characteristics. First, we identified an index acute MI as the patient being admitted to hospital with a primary diagnosis of MI [ICD, Tenth Revision (ICD-10): I21 (Sweden, England, France), I22 (England, France); ICD, Ninth Revision, Clinical Modification (ICD-9-CM): 410.x (excluding 410.x2) (USA)] between 2005 and 2009 (England), 2005 and 2010 (France), 2006 and 2011 (Sweden), and 2002 and 2009 (USA). Where data permitted (England and the USA), the index MI was classified as ST-elevation MI (STEMI) or non-STEMI (NSTEMI) based on MI registry diagnoses (England) or by ICD-9-CM codes (STEMI, 410.0–410.6, 410.8; NSTEMI, 410.7) (USA).^34^ Patients had to have continuous registration in the respective data sets for at least 12 months before the index MI (the first MI admission during the study period). Second, we identified those patients who at 12 months after their index acute MI were alive, with no further MI. We defined the study entry date as 12 months after the date of admission for the index MI. Third, we restricted the population to patients aged 65 years and older at study entry with no upper age bound, because Medicare predominantly covers this age group (the USA has no national unselected sources of data in younger patients).

The study was approved by the Independent Scientific Advisory Committee of the Medicines and Healthcare products Regulatory Agency (protocol number 13_163) in England, regional ethics committee in Linköping, Sweden (reference number 2013/294-31), and Centers for Medicare & Medicaid Services Data Use Agreement in the USA. No ethical approval is required in France for the use of anonymized data.

### Baseline risk factors and co-morbidities

We included demographics (age, sex) and cardiovascular and non-cardiovascular co-morbidities (ICD-9 and ICD-10 codes in Supplementary material online, *Table S1*) appearing as primary or secondary diagnoses in hospital admissions before the study entry date. We considered patients as currently receiving a medication (codes in Supplementary material online, *Table S2*) if their last active prescription or dispensation ended <60 days before study entry. No prescription data were available in the Medicare data. We included percutaneous coronary intervention (PCI) and coronary artery bypass graft (CABG) procedures performed on the day of the index MI up to the following 12 months.

### Endpoints

We studied three outcomes of interest: all-cause death; a composite of death, hospital admission for MI, or hospital admission for stroke; and hospitalized bleeding. The ICD-9/ICD-10 codes used to define these outcomes are shown in Supplementary material online, *Table S3*. Stroke types included ischaemic, haemorrhagic, and unclassified. Hospitalized bleeding was defined as hospital admission with a bleeding cause as a primary diagnosis. Patients were censored at the earliest of experiencing the event of interest (with censoring specific to that event type), deregistration from the primary care practice (England), or end of study period.

### Statistics

Data from each of the four countries were analysed independently following a common protocol. We estimated the direct age- and sex-standardized prevalence of co-morbidities in each country using as reference the 2012 World Health Organization world population truncated to ages 65 years and older. For each country and endpoint, we estimated observed (Kaplan–Meier) and predicted risks, adjusted to the average characteristics of the Swedish patients (aged 78 years, with covariate values shown in Supplementary material online, *Table S4*). We chose Sweden as the reference population because it had the largest sample size. Predicted risks were based on incrementally adjusted Cox models (fitted separately per country): Model 1 included age, sex, and year of index MI; Model 2 included Model 1 covariates plus co-morbidities [history of more than one MI, diabetes, renal disease, heart failure, peripheral arterial disease (PAD), atrial fibrillation, stroke, hospitalized bleeding, chronic obstructive pulmonary disease, and cancer]; Model 3 included Model 2 covariates plus revascularization procedures (CABG or PCI) received in the 12 months following the index MI. Annual risks were estimated as the average annual risks over the first 3 years.

We estimated the relative risks (RRs) for each endpoint in each country and the 95% confidence intervals (CIs) for 3 years of follow-up using as reference the corresponding risks estimated for Sweden. For a time point *t* the RR for country A vs. country B is RR_t=(risk(t)_A)/(risk(t)_B). The overall RR reported is the mean of RR_t{t=0,0.5,…3years}. We verified the proportional hazards assumption of the Cox model within countries by plotting the Schoenfeld residuals and confirmed that RRs did not change with time by plotting time-specific RRs estimated for every half year between 0 and 3 years of follow-up (Supplementary material online, *Figure S5*).

We compared the associations of age, sex, co-morbidities, and revascularization treatments with the outcomes across the different countries based on the adjusted hazard ratios (HRs) in Model 3. The overall mean HR for a risk factor was estimated by combining country-specific HRs via random-effects meta-analysis. For France, risk of hospitalized bleeding was adjusted only for Model 1, owing to the small number of events (*n* = 23). Analyses were performed in R version 15 and SAS version 9.3.

## Results

### Patients

Of the 220 738 patients hospitalized for MI during the study period, 114 364 (54 841 in Sweden, 53 909 in the USA, 4653 in England, and 961 in France) were eligible for inclusion in the analysis (alive, aged 65 years and older, and without subsequent MI at 12-month follow-up; Supplementary material online, *Figure S1*). Median follow-up ranged from 1.5 years (England) to 3.2 years (USA), during which a total of 37 626 deaths, 45 072 events of MI/stroke/death, and 4697 bleeding hospitalizations were observed in the four countries.

### Baseline characteristics

**Figure 1:**
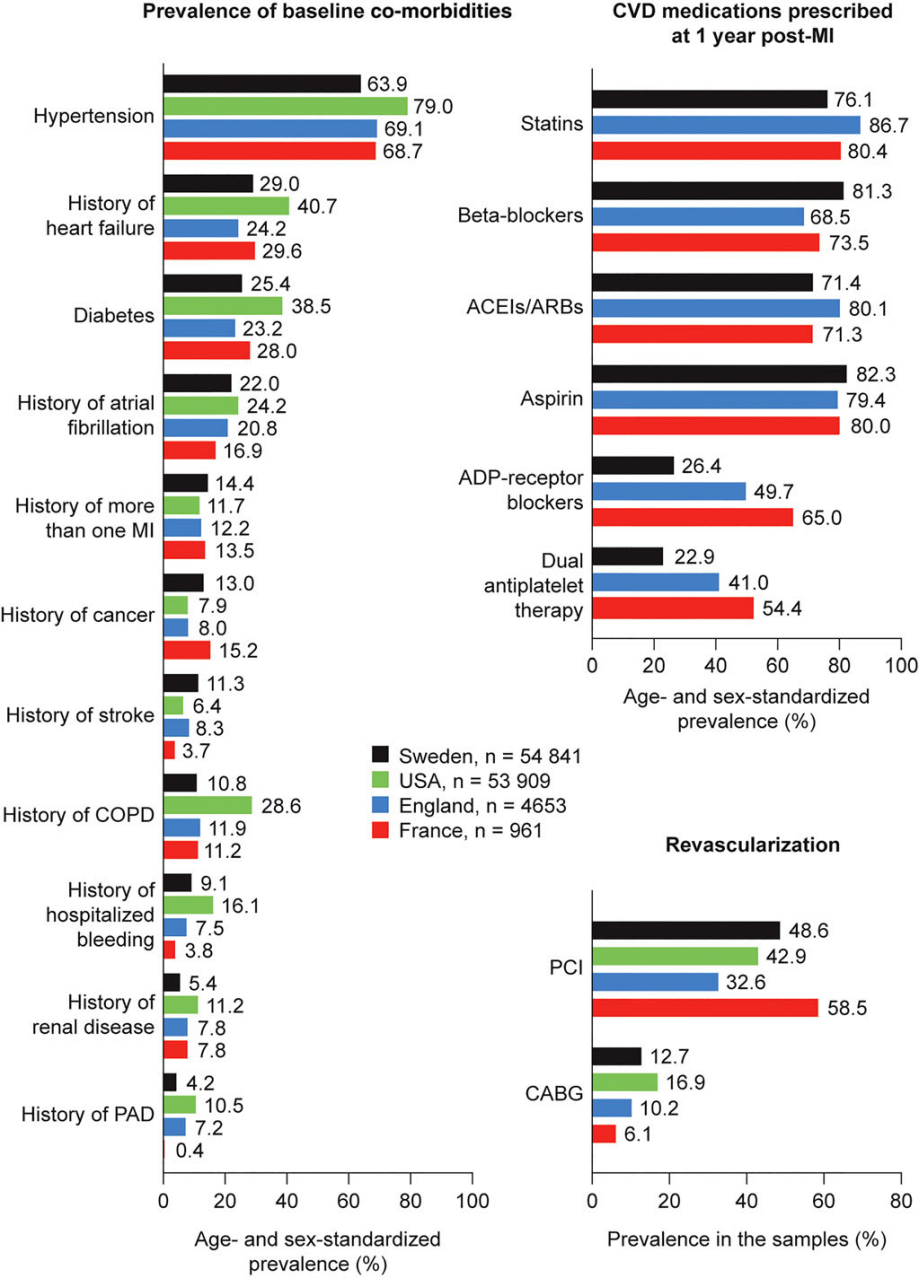
Age- and sex-standardized prevalence of co-morbidities and secondary prevention treatments in post- myocardial infarction survivors aged 65 years and older. Estimates correspond to the direct age- and sex-standardized prevalence of co-morbidities in each country using as reference the 2012 World Health Organization world population truncated to age 65 years and older. ACEI, angiotensin-converting enzyme inhibitor; ADP, adenosine diphosphate; ARB, angiotensin receptor blocker; CABG, coronary artery bypass graft; COPD, chronic obstructive pulmonary disease; CVD, cardiovascular disease; MI, myocardial infarction; PAD, peripheral arterial disease; PCI, percutaneous coronary intervention.

Baseline characteristics of the post-MI survivors from each country are shown in *Table 1*. Mean age ranged from 77.5 years in England to 78.6 years in the USA. After standardization for age and sex, we found that compared with patients from Sweden, England, and France, US patients had a higher prevalence of diabetes, heart failure, PAD, renal disease, and chronic obstructive pulmonary disease, and were more likely to have undergone CABG (*Figure 1*).

**Table 1:** Baseline characteristics for 114 364 myocardial infarction survivors aged 65 years and older in four countries

	Sweden	USA	England	France
Index MI, *n*	80 327	99 343	6653	1308
MI survivor study population, *n* (%)	54 841 (68.3)	53 909 (54.3)	4653 (70.0)	961 (73.5)
Follow-up, years, median (IQR)	2.4 (1.2–3.8)	3.2 (1.6–5.3)	1.5 (0.7–2.5)	3.0 (1.7–3.0)
Demographics				
Women, *n* (%)	23 280 (42.4)	26 524 (49.2)	1933 (41.5)	422 (43.9)
Mean age, years (SD)	78.0 (8.0)	78.6 (7.5)	77.5 (7.7)	77.6 (7.3)
White ethnicity, *n* (%)	Not recorded	48 044 (89.1)	3679 (94.6)	Not recorded
NSTEMI (index MI), *n* (%)	Not recorded	34 576 (64.1)	2393 (51.4)	Not recorded
Mean BMI, kg/m^2^ (SD)	27.5 (4.8)^a^	Not recorded	27.3 (5.0)	Not recorded
Current smoking, *n* (%)	Not recorded	Not recorded	444 (10.3)	Not recorded
Co-morbidities and medical history, *n* (%)				
Diabetes^a^	13 351 (24.3)	18 907 (35.1)	1087 (23.4)	256 (26.6)
>1 MI	8786 (16.0)	6465 (12.0)	651 (14.0)	129 (13.4)
Heart failure	18 170 (33.1)	24 283 (45.0)	1245 (26.8)	319 (33.2)
Cancer	7892 (14.4)	4508 (8.4)	499 (6.9)	167 (17.4)
Atrial fibrillation	13 931 (25.4)	15 215 (28.2)	1152 (24.8)	200 (20.8)
Hypertension	34 689 (63.3)	42 981 (79.7)	3246 (69.8)	663 (69.0)
Stroke	7156 (13.0)	3695 (6.9)	436 (9.4)	45 (4.7)
PAD	2230 (4.1)	5460 (10.1)	353 (7.6)	4 (0.4)
COPD	5478 (10.0)	14 859 (27.6)	556 (11.9)	116 (12.1)
Renal disease	3343 (6.1)	1809 (3.4)	452 (9.7)	99 (10.3)
Dementia	2291 (4.2)	1156 (2.1)	110 (2.4)	49 (5.1)
Previous hospitalized bleeding	5528 (10.1)	9159 (17.0)	398 (8.6)	41 (4.3)
Medication use,^b^*n* (%)				
Aspirin	44 645 (81.4)	Not recorded	3606 (77.5)	723 (75.2)
ADP-receptor blocker	12 741 (23.2)	Not recorded	2357 (50.7)	597 (62.1)
Dual antiplatelet	10 932 (19.9)	Not recorded	1832 (39.4)	469 (48.8)
Statin	38 144 (69.6)	Not recorded	3942 (84.7)	729 (75.9)
β-blocker	43 913 (80.1)	Not recorded	3078 (66.2)	687 (71.5)
ACEIs/ARBs	37 317 (68.0)	Not recorded	3594 (77.2)	667 (69.4)
Calcium channel blocker	12 032 (21.9)	Not recorded	1017 (21.9)	198 (20.6)
Warfarin	5081 (9.3)	Not recorded	408 (8.8)	107 (11.1)
Revascularization (1-year post-index MI), *n* (%)				
CABG	6970 (12.7)	9134 (16.9)	474 (10.2)	59 (6.1)
PCI	26 656 (48.6)	23 099 (42.9)	1519 (32.6)	562 (58.5)

ACEI, angiotensin-converting enzyme inhibitor; ADP, adenosine diphosphate; ARB, angiotensin receptor blocker; BMI, body mass index; CABG, coronary artery bypass graft; COPD, chronic obstructive pulmonary disease; IQR, interquartile range; MI, myocardial infarction; NSTEMI, non-ST-segment-elevation myocardial infarction; PAD, peripheral arterial disease; PCI, percutaneous coronary intervention; SD, standard deviation.

^a^Based on medications (UK, France, Sweden) or diagnosis in primary (UK) or secondary care (UK, Sweden, USA).

^b^Recorded prescription/dispensing or most recent prescription ending <60 days before study entry.

### All-cause death

**Figure 2:**
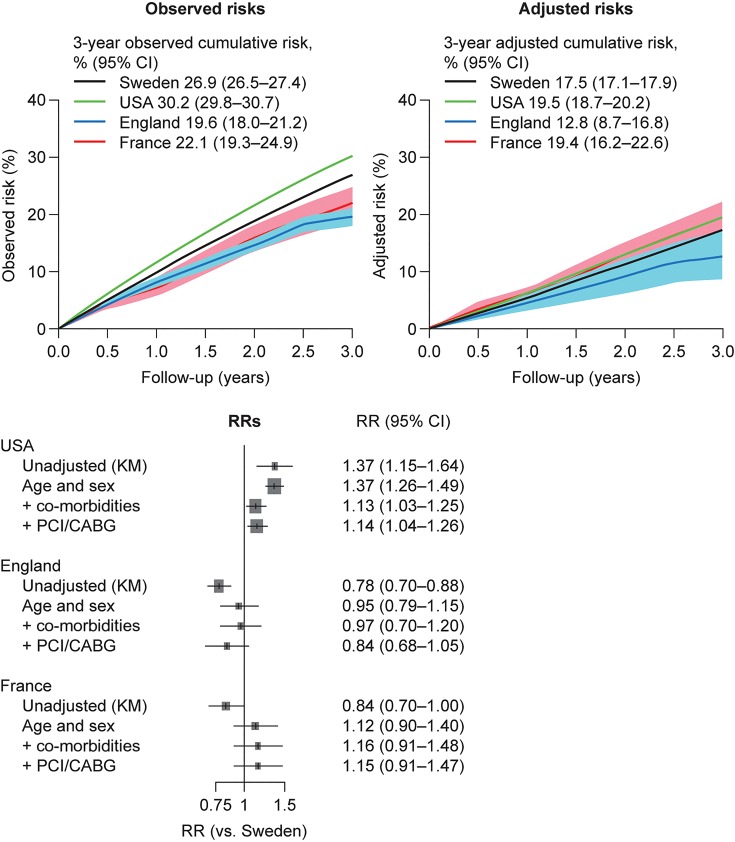
Risks of all-cause death in post-myocardial infarction survivors aged 65 years and older followed from 1 year after the index myocardial infarction. Observed (Kaplan–Meier) risks (top left), adjusted risks (top right), and relative risks vs. Sweden (bottom) in post-myocardial infarction survivors from Sweden (*n* = 54 841), USA (*n* = 53 909), England (*n* = 4653), and France (*n* = 961). CABG, coronary artery bypass graft; CI, confidence interval; KM, Kaplan–Meier; PCI, percutaneous coronary intervention; RR, relative risk.

There were large differences in the unadjusted (Kaplan–Meier) risk of all-cause death across the four countries (*Figure 2*). Event rates remained high throughout follow-up, with fairly constant risks per year. The 3-year cumulative risk of death was lowest in England [19.6% (95% CI, 18.0–21.2)] and France [22.1% (19.3–24.9)], higher in Sweden [26.9% (26.5–27.4)], and highest in the USA [30.2% (29.8–30.7)]. These differences were progressively attenuated to not statistically significant (95% CI for the RR vs. Sweden crossing 1) after sequential adjustments for age, sex, year of index MI, co-morbidities, and revascularization treatments, except for the USA where the RR of death compared with Sweden was slightly higher [RR USA vs. Sweden, 1.14 (95% CI, 1.04–1.26)]. Based on the mean covariates in the Swedish sample as per *Table 1*, the fully adjusted 3-year cumulative risks ranged from 12.8% (England) to 19.5% (USA).

### Myocardial infarction, stroke, and all-cause death

**Figure 3:**
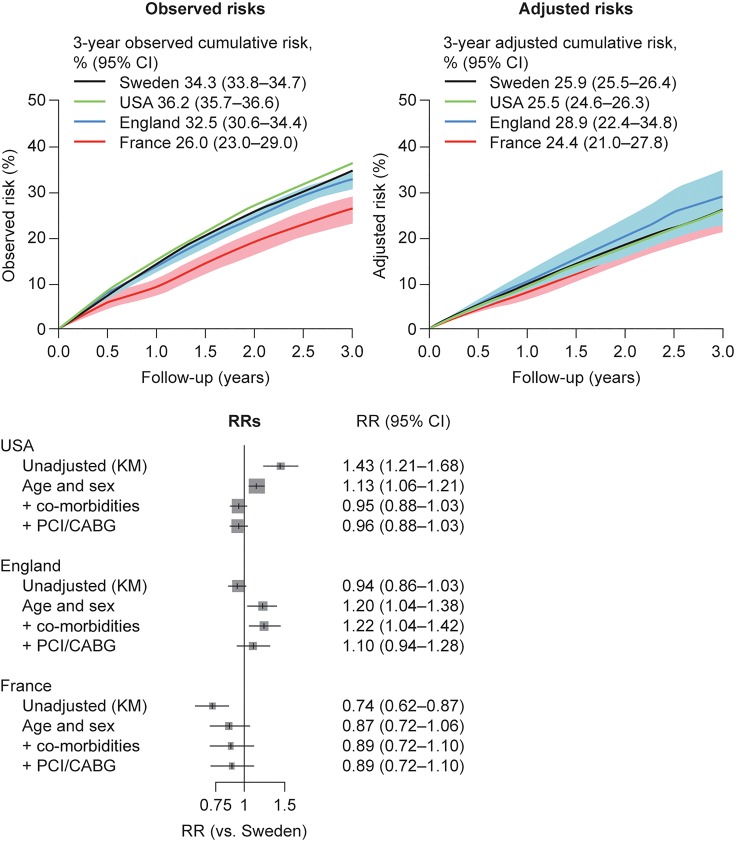
Risks of the composite of myocardial infarction, stroke, and all-cause death in post-myocardial infarction survivors aged 65 years and older followed from 1 year after the index myocardial infarction. Observed (Kaplan–Meier) risks (top left), adjusted risks (top right), and relative risks vs. Sweden (bottom) in post-myocardial infarction survivors from Sweden (*n* = 54 841), USA (*n* = 53 909), England (*n* = 4653), and France (*n* = 961). CABG, coronary artery bypass graft; CI, confidence interval; KM, Kaplan–Meier; PCI, percutaneous coronary intervention; RR, relative risk.

There were large differences in the unadjusted (Kaplan–Meier) risk of the composite endpoint MI, stroke, or death across the four countries (*Figure 3*). Event rates remained high throughout follow-up, with fairly constant risks per year. The lowest risk was observed in France [26.0% (95% CI, 23.0–29.0)] and the highest in the USA [36.2% (95% CI, 35.7–36.6)]; risks were similar in Sweden [34.3% (95% CI, 33.8–34.7)] and England [32.5% (95% CI, 30.6–34.4)]. After adjustments, the risk of MI/stroke/death was similar across all four countries (RRs vs. Sweden were not statistically significant). Based on the mean covariates in the Swedish sample as per *Table 1*, the fully adjusted 3-year cumulative risk of MI, stroke, or death ranged from 24.4% (France) to 28.9% (England).

The proportion of deaths attributed to cardiovascular disease (CVD) was 57.9% (8309/14 341) in Sweden and 46.7% (280/599) in England. English patients had lower observed risks for MI, stroke, or CVD death [23.0% (95% CI, 21.3–24.8)] than Swedish patients [26.1% (95% CI, 25.7–26.5)], but a similar risk after adjustment for age, co-morbidities, and revascularization treatments [RR 0.94 (95% CI, 0.77–1.13)] (Supplementary material online, *Figure S2*).

### Hospitalized bleeding

**Figure 4:**
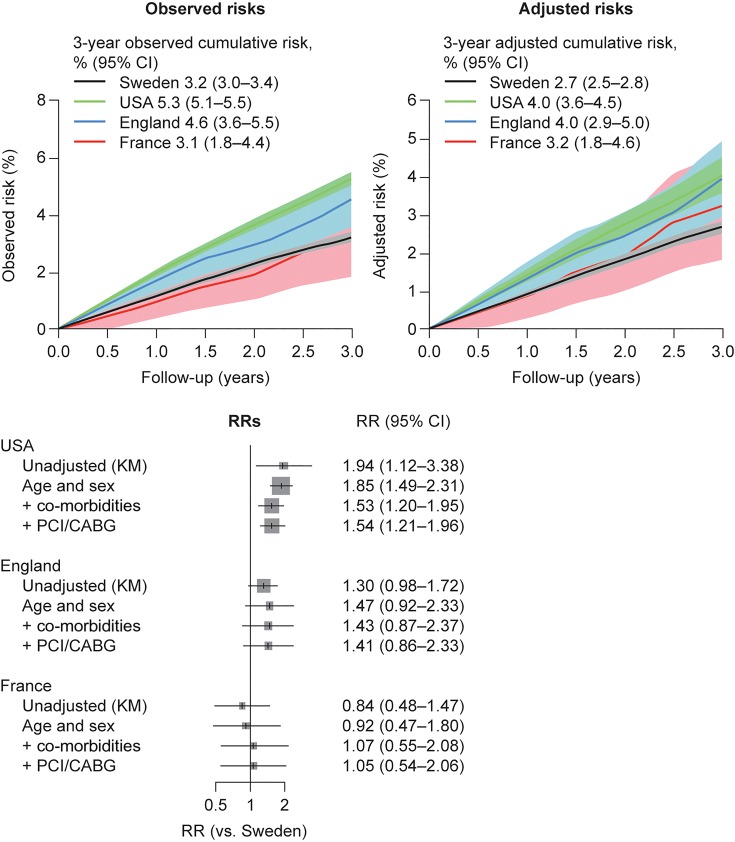
Risks of hospitalized bleeding events in post-myocardial infarction survivors aged 65 years and older followed from 1 year after the index myocardial infarction. Observed (Kaplan–Meier) risks (top left), adjusted risks (top right), and relative risks (bottom) for hospitalized bleeding events among post-myocardial infarction survivors from Sweden (*n* = 54 841), USA (*n* = 53 909), England (*n* = 4653), and France (*n* = 961). CABG, coronary artery bypass graft; CI, confidence interval; KM, Kaplan–Meier; PCI, percutaneous coronary intervention; RR, relative risk.

The observed 3-year cumulative risk of hospitalized bleeding was lowest in France (3.1%) and Sweden (3.2%), higher in England (4.6%), and highest in the USA (5.3%) (*Figure 4*). The adjusted 3-year risk of hospitalized bleeding ranged from 2.7% (Sweden) to 4.0% (USA and England). Compared with Sweden, the fully adjusted RR of bleeding for French and English patients was close to 1.0 (not statistically significant), but was >50% higher for US patients [RR 1.54 (95% CI, 1.21–1.96)].

### Outcome predictors

**Figure 5:**
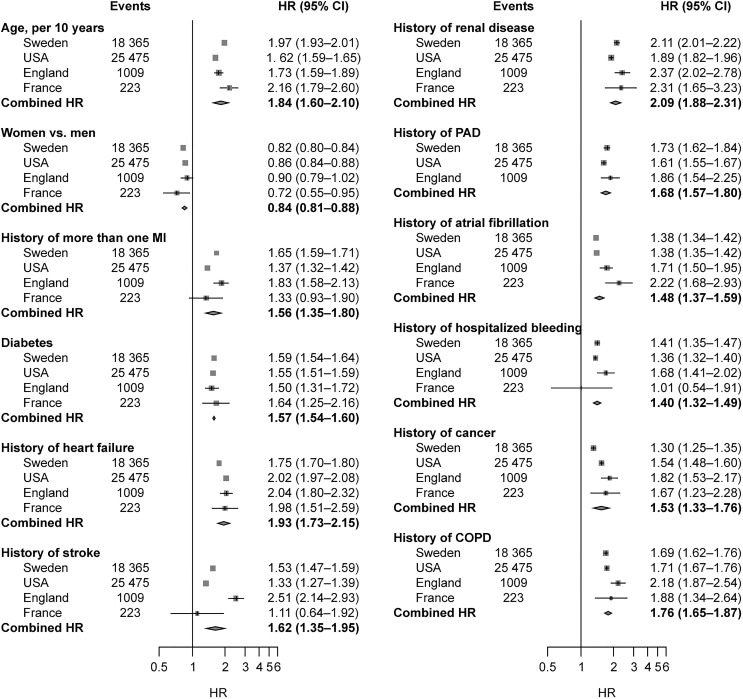
Age- and sex-adjusted hazard ratios (95% confidence interval) for the association of age, sex, and medical history with the composite of myocardial infarction, stroke, and all-cause death among post-myocardial infarction survivors from Sweden (*n* = 54 841), USA (*n* = 53 909), England (*n* = 4653), and France^a^ (*n* = 961). ^a^Incidence of PAD in the French study was <0.5%; hence, it was not possible to obtain estimates of association with outcomes. CI, confidence interval; COPD, chronic obstructive pulmonary disease; HR, hazard ratio; MI, myocardial infarction; PAD, peripheral arterial disease.

Each of the three outcomes showed consistent and strong (majority of HRs >1.5) age- and sex-adjusted associations across the four countries for 12 baseline variables assessed, including risk factors and cardiovascular and non-cardiovascular co-morbidities. The strongest associations (approximately two-fold increase in risk) with the composite of MI, stroke, or death (*Figure 5*) or with all-cause death alone (Supplementary material online, *Figure S3*) were observed for history of renal disease, heart failure, chronic obstructive pulmonary disease, and cancer. For hospitalized bleeding, the strongest associations were observed with history of previous hospitalized bleeding, renal disease, heart disease, PAD, and atrial fibrillation (Supplementary material online, *Figure S4*).

## Discussion

In one of the first US–European uses of hospital record data to evaluate long-term fatal and non-fatal clinical outcomes in CVD, we present two findings that suggest that such data have useful validity and are informative in CVD outcomes research.

First, there was a consistency across all four countries in the high level of risk of further MI, stroke, or death. This occurred in about a third of the patients aged 65 years and above over the next 3 years. This suggests that the high risk is an international phenomenon, rather than a problem with one healthcare system or resulting from the different natures of the underlying record systems. This high risk was considerably higher than that reported in the few smaller previous studies conducted in selected populations,^16^ highlighting the value of examining less-selected patient samples.

Second, there was a consistency across all four countries in the magnitudes of association between 12 baseline risk factors and each of the three disease outcomes. These associations were highly consistent with published findings from smaller, consented studies, supporting the validity of our risk adjustment and comparison of outcomes. Thus, as in previous studies in post-MI survivors,^35–37^ we found strong associations between MI, stroke, or death (with heart failure, stroke, PAD, diabetes, renal disease, and chronic obstructive pulmonary disease) and for hospitalized bleeding (with renal disease, history of hospitalized bleeding, and atrial fibrillation). This provides some evidence of the prognostic validity of the hospital record data coded in different healthcare systems, despite the diversity of data collection systems.

Our approach was to use hospital healthcare records that have features of ‘big data’: being characterized by large sample sizes (‘volume’), diverse data sources, collected for different purposes, and using different coding systems (‘variety’) and lack of researcher control over the meaning of the data (‘veracity’). This approach has been widely advocated in understanding and improving the outcomes of disease,^1^ but seldom applied in international contexts.^38^ The strengths of this approach (compared with voluntary registries or consented studies) lie in direct health system relevance, less bias (larger samples, unselected population-based samples, long-term follow-up with minimal losses), and potential scalability to a wide range of clinical start points and endpoints.^3^ Such record data are also more widely accessible to the research community than those from consented studies.

Our study has important limitations, which are largely inherent in these diverse data sources. First, in only one country (Sweden) were nationwide data accessed; the sample of national data available for research in France was particularly small, but it is, nonetheless, representative of the French population. Second, such health record data will inevitably lack relevant data items. For example, MI subtype (STEMI or NSTEMI) was not recorded across all four countries and could not therefore be included in the model adjustments. However, there is strong evidence that, at 1 year following the index MI, STEMI and NSTEMI shared similar mortality, suggesting that MI subclass is unlikely to have influenced our comparisons.^39^ Information on younger patients, socioeconomic position, ethnicity, drug use, primary care, and cause-specific death was not simultaneously available in all four countries. It is a challenge to these health systems to improve the coverage, depth, and quality of data as part of efforts to expand international comparisons.

We observed an annual risk of death ranging from 6.5% (England) to 10.0% (USA), more than double those in the general population [ranging from 2.9% (France) to 3.7% (UK and USA) in age group 75–79 years] (Supplementary material online, *Table S5*). Since 57.9% of deaths are due to CVD (based on Swedish data), our study population is in the high-risk category based on the 2012 American College of Cardiology/American Heart Association guidelines (where high risk is defined as >3% annual risk of cardiovascular death)^40^ or the 2013 European Society of Cardiology guidelines (where high risk is defined as >3% annual risk of all-cause death).^41^ However, these guidelines are described in the context of the wider population of patients with stable coronary artery disease (many of whom have no history of MI). Also, most of the information comes from meta-analyses of clinical trial data, in which survival is generally higher owing to enrolment of lower-risk populations and better adherence to therapy.

Our finding of higher adjusted death rates and hospitalized bleeding rates in the USA than in Sweden could be artefactual but warrants further investigation. The higher death rates are consistent with the lower life expectancy at age 65 years in the USA compared with Europe (Supplementary material online, *Table S5*).^42^ It is possible that the case mix of patients differs in ways that were not included in our adjustments (e.g. related to the substantially higher prevalence of obesity in the general US population).^42^ We did find that US patients had higher age- and sex-standardized prevalences of diabetes, heart failure, PAD, renal disease, and chronic obstructive pulmonary disease—but each of these factors was included in the risk adjustment models. The USA might also have a higher proportion of ethnic minorities, which could confound between-country comparisons. It is also possible that care differs. Studies in the USA indicate that previously uninsured populations may delay seeking care before becoming eligible for Medicare,^43,44^ and mortality may remain elevated for up to 10 years, compared with those with private insurance.^45^ In contrast, European Union study populations would have had continuous access to healthcare before the age of 65 years.^46^ It is possible that in the USA compared with Europe secondary prevention medications including dual antiplatelet therapy (aspirin and clopidogrel) are used more or at higher doses;^47^ however, evidence of this in unselected populations of MI survivors is lacking. Reported use of other CVD medications in Medicare populations indicates that treatment rates are similar to those observed in the EU study population for β-blockers and calcium channel blockers, but somewhat lower for angiotensin-converting enzyme inhibitors and lipid-lowering therapies.^48–53^

Our findings have clinical implications. First, our results provide evidence for clinicians and regulators when considering new interventions, and when assessing the generalizability of results from clinical trials.^13,43^ The recently reported PEGASUS-TIMI-54 trial results in 1-year MI survivors are the first to demonstrate a role for long-term (i.e. beyond 1 year) dual antiplatelet use.^13^ We applied the trial inclusion and exclusion criteria to our real-world patients (Supplementary material online, *Figure S1*) and demonstrated that the ‘trial-like’ population represents a large proportion (e.g. 66% in Sweden) of the overall MI survivor population, and identified a population at high risk (Supplementary material online, *Figure S6*). Second, our findings suggest the value of considering MI in a chronic-disease management framework, e.g. with a 1-year health check after acute MI optimizing behavioural, secondary preventive, and wider health interventions. We found that a substantial proportion of deaths are from non-cardiovascular causes (53% in England and 42% in Sweden), suggesting the importance of a multidisciplinary team approach in primary care. Guidelines need to be developed for this population that recognize the multitude of cardiovascular co-morbidities (atrial fibrillation, heart failure, diabetes, and PAD) and non-cardiovascular co-morbidities (renal disease, chronic obstructive pulmonary disease) that are highly prevalent among long-term survivors of MI.

In conclusion, analysing hospital record data in the USA and three European countries reveals a consistently high adjusted risk of death, further MI, and stroke in the chronic phase after MI. Inherently, diverse data produced by different health systems may provide insights that are useful in evaluating and comparing the care of patients with chronic diseases and the long-term outcomes of these conditions.

## Supplementary material

Supplementary material is available at *European Heart Journal – Quality of Care and Clinical Outcomes* online.

## Authors' contributions

E.R., M.T., P.B., N.D., M.S., C.E., P. Hasvold, S.J., D.J.C., T.J., N.M., M.J., and H.H.: conceived and designed the research. E.R., E.Y., M.S., P. Hasvold, T.J., and M.J.: acquired the data. E.R., M.T., E.Y., P.B., D.S., and P. Hasvold: performed statistical analysis. E.R., M.S., P. Hasvold, E.J., N.M., and H.H.: handled funding and supervision. M.P.-R.: handled supervision. E.R.: drafted the manuscript. F.T.-D.: was involved in the study design, data interpretation, and review of manuscript. E.R., M.T., E.Y., P.B., P. Hunt, S.-C.C., D.S., M.P.-R., A.T., S.C.D., N.D., C.E., P. Hasvold, E.J., S.J., D.J.C., T.J., N.M., M.J., and H.H.: made critical revision of the manuscript for key intellectual content. E.R., M.T., E.Y., P.B., P. Hunt, S.-C.C., D.S., M.P.-R., A.T., S.C.D., N.D., M.S., F.T.-D., C.E., P. Hasvold, E.J., S.J., D.J.C., T.J., N.M., M.J., and H.H.: given final approval of the submitted manuscript.

## Funding

This study was funded by AstraZeneca, the Medical Research Council Prognosis Research Strategy (PROGRESS) Partnership (H.H., grant G0902393/99558), and by awards to establish the Farr Institute of Health Informatics Research, London from the Medical Research Council, Arthritis Research UK, British Heart Foundation, Cancer Research UK, Chief Scientist Office, Economic and Social Research Council, Engineering and Physical Sciences Research Council, National Institute for Health Research (UK), National Institute for Social Care and Health Research (UK), and Wellcome Trust (E.R., D.S., M.P.-R., and S.D.). S.-C.C. was supported by the Medical Research Population Health Scientist Fellowship (grant MR/M015084/1). T.J. was supported by the Swedish Heart and Lung Foundation. The views expressed in this paper do not necessarily represent the views of the funding bodies. Funding to pay the Open Access publication charges for this article was provided by the Wellcome Trust.

**Conflict of interest:** E.R.: nothing to disclose. M.T.: personal fees from AstraZeneca AB during the conduct of the study; personal fees from AstraZeneca AB outside the submitted work. E.Y.: other from AstraZeneca Pharmaceuticals during the conduct of the study; other from other pharmaceutical consulting clients outside the submitted work: Amgen, Celgene, Takeda, Janssen, Pfizer, and Piramal. P.B.: grants from AstraZeneca during the conduct of the study. P. Hunt: employee of AstraZeneca; other from AstraZeneca Pharmaceuticals during the conduct of the study; other from other pharmaceutical consulting clients outside the submitted work: Roche, Sanofi, Medtronic, Boehringer Ingelheim, and Pfizer. S.-C.C.: nothing to disclose. D.S.: nothing to disclose. M.P.-R.: grants from AstraZeneca during the conduct of the study. A.T.: nothing to disclose. S.C.D.: nothing to disclose. N.D.: grants, personal fees, and non-financial support from Amgen, AstraZeneca, Eli Lilly, and Sanofi; grants and personal fees from Bayer, Daiichi Sankyo, and MSD; personal fees and non-financial support from Servier; personal fees from GSK, Novartis, Novo-Nordisk, Pfizer, Roche, and Boehringer Ingelheim; during the conduct of the study. M.S.: other from AstraZeneca during the conduct of the study. F.T.-D.: employee of AstraZeneca. C.E.: employee of AstraZeneca. P. Hasvold: other from AstraZeneca during the conduct of the study; other from AstraZeneca outside the submitted work; personal fees from AstraZeneca during the conduct of the study; personal fees from AstraZeneca outside the submitted work. E.J.: employee of AstraZeneca. S.J.: employee of AstraZeneca. D.J.C.: grants and personal fees from AstraZeneca and Eli Lilly; grants from Daiichi Sankyo; outside the submitted work. T.J.: the APOLLO-project is financed by AstraZeneca. N.M.: grants and personal fees from AstraZeneca during the conduct of the study; grants from most pharma companies outside the submitted work. M.J.: personal fees from SanofiAventis and AstraZeneca outside the submitted work. H.H.: grants from AstraZeneca during the conduct of the study.

## Supplementary Material

Supplementary Tables
