# Supplementary material for: Using big data from health records from four countries to evaluate chronic disease outcomes: a study in 114 364 survivors of myocardial infarction
Source: Eur Heart J Qual Care Clin Outcomes. 2016 Feb 15;2(3):172–83. doi: 10.1093/ehjqcco/qcw004 (PMC5815620; doi:10.1093/ehjqcco/qcw004)
Supplement: Supplementary Tables [file qcw004_Supplementary_Data.zip › qcw004supp.docx]

# Supplementary material

# Using big data from health records from four countries to evaluate chronic disease outcomes: a study in 114 364 patients after myocardial infarction

Eleni Rapsomaniki, Marcus Thuresson, Erru Yang, Patrick Blin, Phillip Hunt, Sheng-Chia Chung, Dimitris Stogiannis,
Mar Pujades-Rodriguez, Adam Timmis, Spiros Denaxas, Nicolas Danchin, Michael Stokes, Florence Thomas-Delecourt,
Cathy Emmas, Pål Hasvold, Em Jennings, Saga Johansson, David J. Cohen, Tomas Jernberg, Nicholas Moore,
Magnus Janzon, Harry Hemingway

**Methods**

**Table S1** Comorbidity definitions based on ICD-9 codes (used in the Swedish and US studies) and ICD-10 codes (used in the Swedish, English, and French studies)

**Table S2** Medication codes

**Table S3** Endpoint definitions based on ICD-9 codes (used in the Swedish and US studies) and ICD-10 codes (used in the Swedish, English, and French studies)

**Table S4** Covariate values used in model adjustments, corresponding to the average baseline characteristics in the Swedish study

**Results**

**Figure S1** Study sample definition from the time of acute MI and in relation to PEGASUS TIMI-54 trial inclusion and exclusion criteria in each country

**Figure S2** Observed and adjusted risks of the composite of MI, stroke, and fatal CVD among post-MI survivors from Sweden (n = 54 841) and England (n = 4653)

**Figure S3** Age- and sex- adjusted hazard ratios (95% CI) for the association of age, sex, and medical history with all-cause death among post-MI survivors from Sweden (n = 54 841), USA (n = 53 909), England (n = 4653), and France (n = 961)

**Figure S4** Age- and sex- adjusted HRs (95% CI) for the association of age, sex, comorbidity, and medical history with hospitalized bleeding in post-MI survivors from Sweden (n = 54 841), USA (n = 53 909), and England (n = 4653)

**Figure S5** Plots of relative risks (vs. Sweden) at different time points between 0.5 and 3 years of follow-up based on fully adjusted risks (age, sex, comorbidity, and revascularization).

**Figure S6** Three-year observed risks (Kaplan–Meier) of all-cause death, composite of MI, stroke and death, composite of MI, stroke and fatal CVD, and hospitalized bleeding for patients meeting PEGASUS TIMI-54 inclusion and exclusion criteria

**Contextual information about each country**

**Table S5** Population health profile and healthcare comparison among participant countries in 2010

This supplementary material has been provided by the authors to give readers additional information about their work.

**Table S1 Comorbidity definitions based on ICD-9 codes (used in the Swedish and US studies) and ICD-10 codes (used in the Swedish, English, and French studies)**

| **Condition^a^** | **ICD-10 codes (ICD-9 equivalent)** | |
| --- | --- | --- |
| Hypertension | I10–I15 (401.x, 405.x) | Essential and secondary hypertension |
| Heart failure | I11.0 (402.01, 402.11, 402.91) | Hypertensive heart disease with (congestive) heart failure |
|  | I130 (404.01, 404.11, 404.91) | Hypertensive heart and renal disease with (congestive) heart failure |
|  | I132 (404.03, 404.13, 404.93) | Hypertensive heart and renal disease with both (congestive) heart failure and renal failure |
|  | I26.0 (415.0) | Pulmonary embolism with mention of acute cor pulmonale |
|  | I50 (428.xx) | Heart failure |
| Atrial fibrillation | 148 (427.31) | Atrial fibrillation and flutter |
| Peripheral arterial disease | I73.1 (443.1) | Thromboangiitis obliterans (Buerger) |
|  | I73.8 (443.89) | Other specified peripheral vascular diseases |
|  | I73.9 (443.9) | Peripheral vascular disease, unspecified |
|  |  |  |
| Cancer | C00-C99 (140–239.x) | Malignant neoplasms |
| Dementia | F00 (331.0) | Dementia in Alzheimer’s disease |
|  | F01 (290.4X) | Vascular dementia |
|  | F02 (294.10) | Dementia in other diseases classified elsewhere |
|  | F03 (290.0–290.3, 290.8, 290.9) | Unspecified dementia |
|  | F051 (293.0, 293.1) | Delirium superimposed on dementia |
| COPD | J40 (490.xx) | Bronchitis, not specified as acute or chronic |
|  | J41 (491.0, 491.1, 491.8) | Simple and mucopurulent chronic bronchitis |
|  | J42 (491.9) | Unspecified chronic bronchitis |
|  | J43 (492.0, 492.8) | Emphysema |
|  | J44 (491.22, 493.21, 491.21, 493.22, 491.20, 493.20, 496.xx) | Other COPD |
|  | J47 (494.1, 494.0) | Bronchiectasis |
| Renal disease (moderate/severe) | N01 (580.4) | Rapidly progressive nephritic syndrome |
|  | N03 (582.0–582.4) | Chronic nephritic syndrome |
|  | N05.2 (583.1) | Unspecified nephritic syndrome; diffuse membranous glomerulonephritis |
|  | N05.3 (583.2) | Unspecified nephritic syndrome; diffuse mesangial proliferative glomerulonephritis |
|  | N05.4 (583.2) | Unspecified nephritic syndrome; diffuse endocapillary proliferative glomerulonephritis |
|  | N05.5 (583.2) | Unspecified nephritic syndrome; diffuse mesangiocapillary glomerulonephritis |
|  | N05.6 (583.89) | Unspecified nephritic syndrome; dense deposit disease |
|  | N07.2 (583.1) | Hereditary nephropathy, not elsewhere classified; diffuse membranous glomerulonephritis |
|  | N07.3 (583.2) | Hereditary nephropathy, not elsewhere classified; diffuse mesangial proliferative glomerulonephritis |
|  | N07.4 (583.2) | Hereditary nephropathy, not elsewhere classified; diffuse endocapillary proliferative glomerulonephritis |
|  | N18.3 (585.3) | Chronic kidney disease, stage 3 |
|  | N18.4 (585.4) | Chronic kidney disease, stage 4 |
|  | N18.5 (585.5) | Chronic kidney disease, stage 5 |
|  | N18.9 (585.9) | Chronic kidney disease, unspecified |
|  | N19 (586) | Unspecified kidney failure |
|  | N25 (588) | Disorders resulting from impaired renal tubular function |

^a^Primary/secondary diagnosis.

COPD, chronic obstructive pulmonary disease; ICD, International Classification of Diseases.

**Table S2 Medication codes**

|  | **Sweden**  **(ATC codes)** | **England**  **(BNF codes)** | **France**  **(ATC codes)** |
| --- | --- | --- | --- |
| Clopidogrel | B01AC04 | BNF 2.9, substance clopidogrel | B01AC04  B01AC30 |
| Prasugrel | B01AC22 | BNF 2.9,  substance prasugrel | B01AC22 |
| Aspirin | B01AC06 | BNF 2.9,  substance aspirin | B01AC06  C10BX01  C10BX02  C10BX04  C10BX05  B01AC30 |
| Statins | C10AA | BNF 2.12 | C10AA  C10BA  C10BX |
| Beta-blockers | C07 | BNF 2.4 | C07 |
| Calcium-channel blockers | C08 | BNF 2.6.2 | C08 |
| ACEIs or ARBs | C09 | BNF 2.5.5.1  BNF 2.5.5.2 | C09 |
| Diabetes medication | A10 | BNF 6.1.1  BNF 6.1.2 | A10 |

ACEI, angiotensin-converting enzyme inhibitor; ARB, angiotensin II receptor blocker; ATC, Anatomical Therapeutic Chemical; BNF, British National Formulary.

**Table S3 Endpoint definitions based on ICD-9 codes (used in the Swedish and US studies) and ICD-10 codes (used in the Swedish, English, and French studies)**

| **Condition**^a^ | **ICD-10 codes** | **ICD-9 equivalent** | **Acute MI** |
| --- | --- | --- | --- |
| MI | I21 xx | 410.xx [excluding 410.x2] | Acute MI |
|  | I22 xx |  | Subsequent MI |
| Stroke | I60 (I600–I609) | 430 | Subarachnoid haemorrhage |
|  | I61 (I610–I619) | 431 | Intracerebral haemorrhage |
|  | I62 (I620, I621, I 629) | 432.0, 432.1, 432.9 | Other non-traumatic intracranial haemorrhage |
|  | I63 (I631–I639) | 433.x1, 434.x1, 436.xx | Cerebral infarction |
|  | I64 |  | Stroke, not specified as haemorrhage or infarction |
| CVD mortality | I00–I99 |  |  |
| Bleeding | Bleeding in the brain | | |
|  | I60 | 430 | Subarachnoid haemorrhage |
|  | I61 | 431 | Intracerebral haemorrhage |
|  | I62 | 432.0, 432.1, 432.9 | Other non-traumatic intracranial haemorrhage |
|  | Gastrointestinal bleeding | | |
|  | K25.0 | 531.00, 531.01 | Gastric ulcer; acute with haemorrhage |
|  | K25.2 | 531.20, 531.21 | Gastric ulcer; acute with both haemorrhage and perforation |
|  | K25.4 | 531.40, 531.41 | Gastric ulcer; chronic or unspecified with haemorrhage |
|  | K25.6 | 531.60, 531.61 | Gastric ulcer; chronic or unspecified with both haemorrhage and perforation |
|  | K26.0 | 532.00, 532.01 | Duodenal ulcer; acute with haemorrhage |
|  | K26.2 | 532.20, 532.21 | Duodenal ulcer; acute with both haemorrhage and perforation |
|  | K26.4 | 532.40, 532.41 | Duodenal ulcer; chronic or unspecified with haemorrhage |
|  | K26.6 | 532.60, 532.61 | Duodenal ulcer; chronic or unspecified with both haemorrhage and perforation |
|  | K27.0 | 533.00, 533.01 | Peptic ulcer, site unspecified; acute with haemorrhage |
|  | K27.2 | 533.20, 533.21 | Peptic ulcer, site unspecified; acute with both haemorrhage and perforation |
|  | K27.4 | 533.40, 533.41 | Peptic ulcer, site unspecified; chronic or unspecified with haemorrhage |
|  | K27.6 | 533.60, 533.61 | Peptic ulcer, site unspecified; chronic or unspecified with both haemorrhage and perforation |
|  | K28.0 | 534.00, 534.01 | Gastrojejunal ulcer; acute with haemorrhage |
|  | K28.2 | 534.20, 534.21 | Gastrojejunal ulcer; acute with both haemorrhage and perforation |
|  | K28.4 | 534.40, 534.41 | Gastrojejunal ulcer; chronic or unspecified with haemorrhage |
|  | K28.6 | 534.60, 534.61 | Gastrojejunal ulcer; chronic or unspecified with both haemorrhage and perforation |
|  | K29.0 | 535.01 | Acute haemorrhagic gastritis |
|  | K62.5 | 569.3 | Haemorrhage of anus and rectum |
|  | K92.0 | 578.0 | Haematemesis |
|  | K92.1 | 578.1 | Melaena |
|  | K92.2 | 578.9 | Gastrointestinal haemorrhage, unspecified |
|  | Other location | |  |
|  | H35.6 | 362.81 | Retinal haemorrhage |
|  | H43.1 | 379.23 | Vitreous haemorrhage |
|  | H45.0 | NA | Vitreous haemorrhage in diseases classified elsewhere |
|  | R04.1 | 784.8 | Haemorrhage from throat |
|  | R04.8 | 786.39 | Haemorrhage from other sites in respiratory passages |
|  | R04.9 | 786.3 | Haemorrhage from respiratory passages, unspecified |

^a^Principal diagnosis as outcome, principal/secondary diagnosis to record history of disease.
CVD, cardiovascular disease; ICD, International Classification of Diseases; IHD, ischaemic heart disease; MI, myocardial infarction; NA, not available.

**Table S4 Covariate values used in model adjustments (corresponding to the average baseline characteristics in the Swedish study)**

| **Characteristic** | **Value** |
| --- | --- |
| Age, years | 78.0 |
| Female sex | 42.4% |
| Year of index MI | 2008 |
| Hypertension | 63.3% |
| Diabetes mellitus | 24.3% |
| History of more than one1 MI | 16.0% |
| History of heart failure | 33.1% |
| History of PAD | 4.1% |
| History of renal disease | 6.1% |
| History of stroke | 13.0% |
| History of atrial fibrillation | 25.4% |
| History of hospitalized bleeding | 10.1% |
| History of cancer | 14.4% |
| History of COPD | 10.0% |
| CABG | 12.61% |
| PCI | 55.80% |

CABG, coronary artery bypass graft; COPD, chronic obstructive pulmonary disease; MI, myocardial infarction; PAD, peripheral arterial disease; PCI, percutaneous coronary intervention.

**Figure S1** Study sample definition from the time of acute MI and in relation to PEGASUS TIMI-54 trial inclusion and exclusion criteria in each country.

**220 738 acute MI patients**

Sweden: n = 108 315

USA: n = 99 343

England: n = 10 854

France: n = 2226

79 851 either died or had a further MI in the 1 year following the index admission

**140 887 alive at first anniversary of the acute MI**

Sweden: n = 77 976

USA: n = 53 909

England: n = 7238

France: n = 1764

PEGASUS-TIMI-54-LIKE POPULATION

**MAIN ANALYSIS POPULATION**

19 126 did not meet further trial inclusion criteria^a^

15 180 met trial exclusion criteria^b^

26 523 aged <65 years at first anniversary of the acute MI

N = 106 581

*PEGASUS-TIMI-54-like*

*post-MI survivors*

Sweden: n = 51 298

USA: n = 50 052

England: n = 4290

France: n = 941

**N = 114 364 post-MI survivors**

**aged 65 years** **or older**

**Sweden: n=54 841**

**USA: n=53 909**

**England: n=4653**

**France: n=961**

^a^PEGASUS-TIMI-54 trial inclusion criteria were defined as any of the following: age 65 years or older, a second prior MI, diabetes mellitus, chronic renal dysfunction.^c^

^b^Trial exclusion criteria were history of stroke, on dialysis pre-/post-index MI, use of oral anticoagulants within 30 days of the first anniversary of the acute MI.^c^

^c^Medical history of more than one1 MI, renal disease and stroke are based on hospital admissions (primary/secondary diagnoses) up to the date of entry to the survivor cohort (1 year after the index MI).

**Figure S2** Observed and adjusted risks of the composite of MI, stroke, and fatal CVD among post-MI survivors from Sweden (n = 54 841) and England (n = 4653).

**
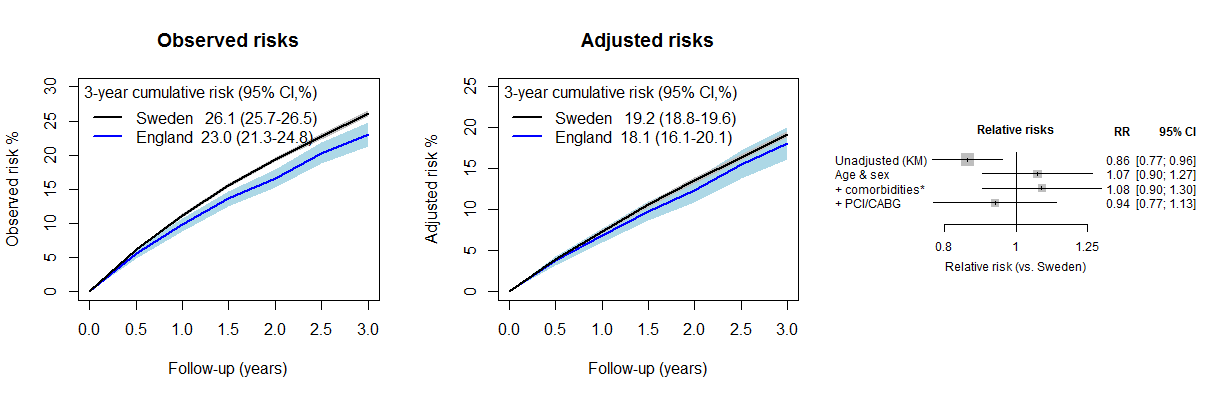
**

Left panel: observed (Kaplan–Meier) risks. Right panel: relative risks using as reference the corresponding risks in the Swedish study between 0 and 3 years of follow-up.

*History of more than one MI, hypertension, renal disease, heart failure, PAD, stroke, atrial fibrillation, hospitalized bleeding, cancer, or COPD; CABG or PCI received in the 12 months following the index MI.

All models are adjusted for year of index MI.

CABG, coronary artery bypass graft; CI, confidence interval; COPD, chronic obstructive pulmonary disease; CVD, cardiovascular disease; KM, Kaplan–Meier; MI, myocardial infarction; PAD, peripheral arterial disease; PCI, percutaneous coronary intervention; RR, relative risk.

**Figure S3** Age- and sex- adjusted hazard ratios (95% CI) for the association of age, sex, and medical history with all-cause death among post-MI survivors from Sweden (n = 54 841), USA (n = 53 909), England (n = 4653), and France^a^ (n=961).


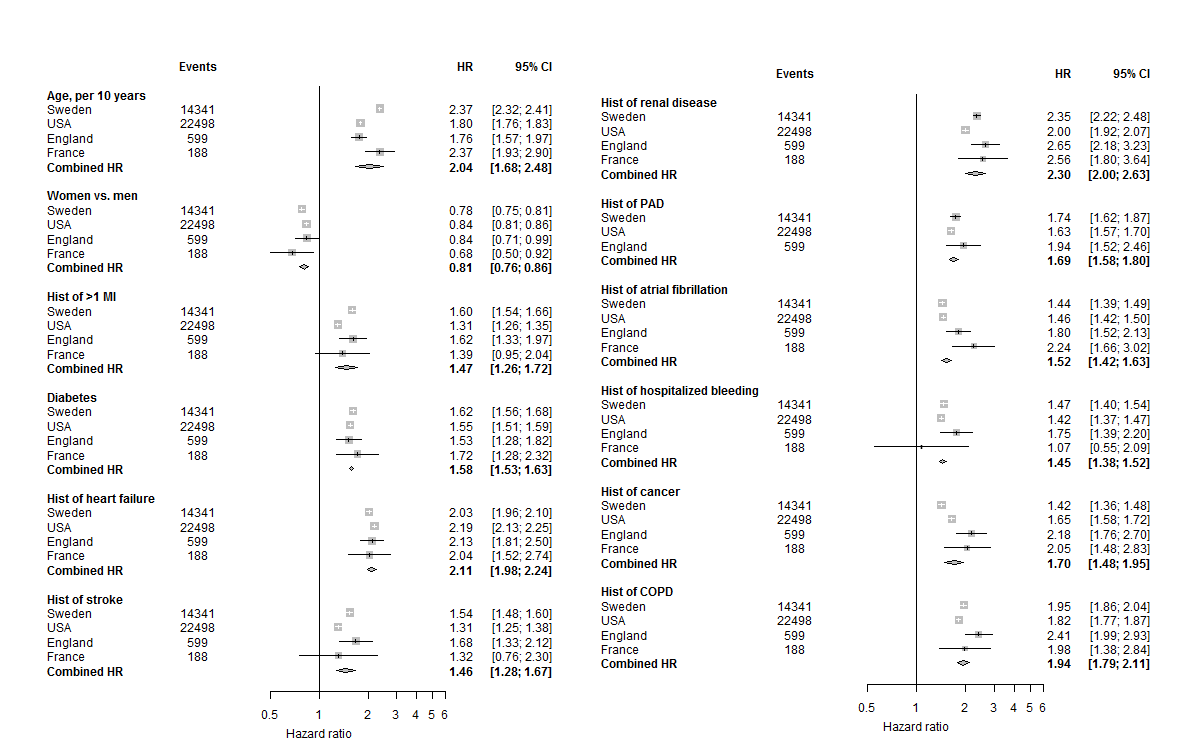


CI, confidence interval; COPD, chronic obstructive pulmonary disease; HR, hazard ratio; MI, myocardial infarction; PAD, peripheral arterial disease.

^a^Incidence of PAD in the French study was <0.5%; hence, it was not possible to obtain estimates of association with outcomes.

**Figure S4** Age- and sex- adjusted HRs (95% CI) for the association of age, sex, comorbidity, and medical history with hospitalized bleeding in post-MI survivors from Sweden (n = 54 841), USA (n = 53 909), and England (n = 4653)^a^


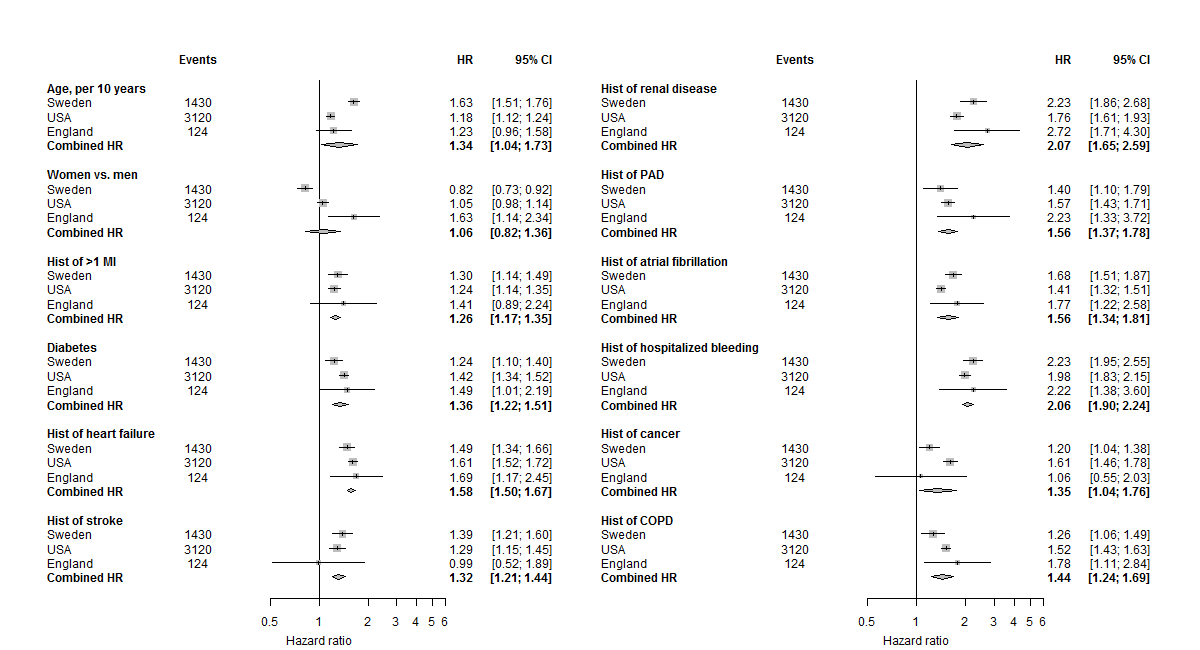


^a^There were too few events (n = 23) to estimate the associations of comorbidities with hospitalized bleeding in the French sample.

CI, confidence interval; COPD, chronic obstructive pulmonary disease; HR, hazard ratio; MI, myocardial infarction; PAD, peripheral arterial disease.

**Figure S5** Plots of relative risks (vs. Sweden) after 0.5 to 3 years of follow-up based on fully adjusted risks (age, sex, comorbidity, and revascularization).

**Figure S6 Three-year observed risks (Kaplan–Meier) of all-cause death; composite of MI, stroke, and death; composite of MI, stroke, and fatal CVD; and hospitalized bleeding for patients meeting PEGASUS TIMI-54 inclusion and exclusion criteria**


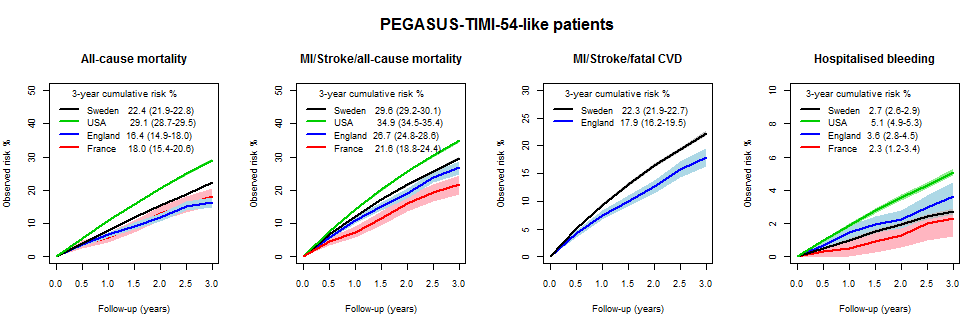


Number of patients meeting PEGASUS TIMI-54 inclusion and exclusion criteria: Sweden, n = 51 298; USA, n = 50 052; England, n = 4290; France, n = 941 (see Figure S1).

CI, confidence interval; CVD, cardiovascular disease; MI, myocardial infarction.

**Table S5 Population health profile and healthcare comparison in Sweden, USA, UK, and France in 2010^a^**

|  | **Sweden** | **USA** | **UK** | **France** |
| --- | --- | --- | --- | --- |
| **Sociodemographics** | | | | |
| Population size, millions | 9.3 | 309.3 | 62.8 | 64.9 |
| Aged 65 years and older, % | 18 | 13.1 | 16.2 | 16.8 |
| Living in poverty,^b^ % | 17 | 24 | 17 | 14 |
| Gross domestic income per capita, $ | 49 377 | 48 358 | 36 573 | 39 573 |
| **Healthcare system indicators** | | | | |
| Gross domestic product spent on health, % | 9.5 | 17.0 | 9.4 | 11.6 |
| Hospital beds, per 1000 population | 2.8 | 3.1 | 3.3 | 6.4 |
| **Population health profile** | | | | |
| Life expectancy of men at age 65, years | 18.3 | 17.7 | 18.2 | 18.9 |
| Life expectancy of women at age 65, years | 21.2 | 20.3 | 20.8 | 23.4 |
| Daily smokers, % | 13.6 | 15.1 | 19.6 | 23.3 |
| Alcohol consumption, litres per capita | 7.3 | 8.6 | 10.3 | 12.0 |
| **Annual all-cause mortality** | | | | |
| 65–69 years | 1.2 | 1.5 | 1.3 | 1.1 |
| 70–74 years | 1.9 | 2.3 | 2.2 | 1.7 |
| 75–79 years | 3.4 | 3.7 | 3.7 | 2.9 |
| 80–84 years | 6.2 | 6.1 | 6.5 | 5.2 |
| **Care and outcome of coronary disease** | | | | |
| Transluminal coronary angioplasty inpatient cases per 100 000 population (2009) | 171.9 | 193.3 | 92.1 | 193.6 |
| Coronary artery bypass graft inpatient cases per 100 000 population (2009) | 42.0 | 79.0 | 32.9 | 30.2 |
| Age (sex)-standardized AMI 30-day in-hospital mortality per 100 hospital discharges | 4.8 | 5.5 | 8.4 | 6.2 |

^a^OECD. StatExtracts: <http://stats.oecd.org/> (Accessed 24 July 2014).

^b^Defined as 60% of national median household disposable income after taxes and transfers.

AMI, acute myocardial infarction.
